# External Dacryocystorhinostomy for the Treatment of Functional Nasolacrimal Drainage Obstruction

**DOI:** 10.4274/tjo.24381

**Published:** 2015-10-05

**Authors:** İlke Şimşek, Özge Yabaş Kızıloğlu, Şule Ziylan

**Affiliations:** 1 Medicine Hospital, Clinic of Ophthalmology, İstanbul, Turkey; 2 Bahçeşehir University Faculty of Medicine, Göztepe Medical Park Hospital, Clinic of Ophthalmology, İstanbul, Turkey; 3 Yeditepe University Faculty of Medicine, Department of Ophthalmology, İstanbul, Turkey

**Keywords:** Functional nasolacrimal drainage obstruction, external dacryocystorhinostomy, lacrimal scintigraphy

## Abstract

**Objectives:**

To determine the outcome and long-term efficacy of external dacryocystorhinostomy (ext-DCR) with or without bicanalicular silicon intubation in patients with functional nasolacrimal drainage obstruction (FNLDO).

**Materials and Methods:**

Patients with epiphora and patent lacrimal systems on nasolacrimal irrigation were prospectively enrolled in the study. Each patient was assessed with lacrimal scintigraphy to differentiate drainage abnormalities as presac (proximal) or postsac (distal) delays. All patients underwent ext-DCR; bicanalicular silicone intubation was performed only in presac delay cases. On follow-up examinations patients were asked to report their symptoms as none, mild, moderate or unchanged. Success was defined as lacrimal patency to irrigation and no or mild epiphora at the end of the follow-up period.

**Results:**

Twenty-six lacrimal systems of 23 patients were eligible for inclusion. There were 9 presac delay and 17 postsac delay cases. Average follow-up time was 72.85 weeks (47-88 weeks). A successful outcome was achieved in 76.9% of the operated lacrimal systems. Success rate was 55.5% among presac obstructions and 88.2% among postsac obstructions.

**Conclusion:**

The long-term efficacy of ext-DCR in FNLDO patients is confirmed with our overall successful outcome of 76.9%. In preoperative assessment, lacrimal scintigraphy is helpful to determine the surgical approach and to predict the surgical outcome.

## INTRODUCTION

Epiphora is a common condition in ophthalmological practice, a result of decreased tear drainage. Inadequate tear drainage is either caused by a pump failure or an anatomical obstruction along the lacrimal drainage pathway. There is a subgroup of patients with epiphora exhibiting decreased tear drainage and increased tear line associated with normal pump function and a patent lacrimal system on nasolacrimal irrigation. This status can be termed “functional nasolacrimal drainage obstruction (FNLDO)”. Lacrimal scintigraphy is helpful in assessing these patients with functional epiphora. The level of the functional obstruction can be demonstrated with this non-invasive test.^1,2,3,4,5,6,7^

There are several treatment options for FNLDO, including silicone intubation, balloon catheter dilatation and external dacryocystorhinostomy. Bicanalicular or monocanalicular silicone intubations have successful outcomes in 53 to 60% of FNLDO cases.^8,9,10,11,12,13^ Balloon catheter dilatation with and without intubation has similar success rates ranging between 53 and 68%.^13,14,15,16,17,18^

The outcome of external dacryocystorhinostomy (ext-DCR) surgery in patients with FNLDO is somewhat uncertain, with reported success rates ranging from 50 to 94%.^3,4,19,20,21,22,23,24^ In this study, we aimed to determine the outcomes and long-term efficacy of ext-DCR with or without bicanalicular silicone intubation in a group of patients with FNLDO.

## MATERIALS AND METHODS

Patients who were clinically diagnosed with unilateral or bilateral FNLDO were prospectively enrolled in the study and data were collected between December 2005 and September 2012. Informed consent was obtained from each patient before enrollment and this study was approved by the Ethics Committee. Patients with epiphora who had normal lid position and tonus, adequate puncta, normal nasal examination and patent lacrimal systems with nasolacrimal irrigation were included. Exclusion criteria included pump failure, ocular surface disease, dry eye syndrome, trichiasis, distichiasis, and eyelid margin diseases like blepharitis or meibomitis. Patients with a history of radioactive iodine therapy, chemotherapy or radiotherapy, and patients who had previous trauma to the lacrimal region or who had granulomatous or inflammatory diseases like sarcoidosis were also excluded from the study.

Our technique for nasolacrimal irrigation involved the use of a topical anaesthetic before introducing a lacrimal cannula into the lower punctum. Normal saline was irrigated using a 2 ml syringe and the lacrimal system was regarded as freely patent if there was minimal or no regurgitation at the punctum and ready flow of fluid which the patient confirmed as saline into the throat.

Patients were subsequently investigated with a standardised lacrimal scintigraphy. This required the patient to be sitting upright in front of the pinhole collimator of a gamma camera. A drop of technetium-99m pertechnetate was placed into the inferior fornix of both eyes and the patient was requested to remain still, but to blink normally. A dynamic study was performed initially, with the tracer distribution imaged every 10 seconds for the first 160 seconds. After lacrimal sac massage, static views were then taken routinely at 5, 10, 15, and 20 minutes. Using this information, preoperative lacrimal scintigraphy designated drainage abnormalities as presac or postsac delays. A presac delay was diagnosed if tracer failed to reach the sac by the end of the dynamic phase. A postsac delay was diagnosed if there was early filling of the sac and the sac retained full contrast by the end of the study.

All included patients underwent ext-DCR, which was routinely performed under general anesthesia by the same oculoplastic surgeon. Surgery involved complete opening of the lacrimal sac and suturing of anterior and posterior flaps. With ext-DCR, bicanalicular silicone intubation (product #5012, Visitec, Sarasota, Florida, USA) was performed only in cases with presac delay on lacrimal scintigraphy.

Postoperative routine topical chloramphenicol and fluorometholone eye drops four times a day for one week were prescribed. Patients were asked to return for regular follow-up examinations in the first week, first month, and sixth month after the operation. Follow-up examinations continued at six-month intervals thereafter until the end of the study. Patients were asked to subjectively report their symptoms as none, mild, moderate or unchanged at each visit. Success was defined as lacrimal patency to irrigation and no or mild epiphora at the last follow-up visit.

## RESULTS

Baseline characteristics of the 23 patients (26 operated lacrimal systems) are shown in Table 1. According to the lacrimal scintigraphy results, there were 9 cases with presac delay and 17 with postsac delay. Ext-DCR with bicanalicular silicone intubation was performed on the former cases, whereas only ext-DCR was performed on the latter. No major intraoperative or postoperative complications were observed. The average time for removal of the silicone tubes was 16 weeks (range, 11 to 30 weeks) and the average follow-up duration was 72.85 weeks (range, 47-88 weeks).

An overall successful outcome of no or mild symptoms was achieved in 76.9% of the operated lacrimal systems (55.5% in presac obstructions, 88.2% in postsac obstructions). Among the 6 unsuccessful cases, 4 had presac and 2 had postsac delay on lacrimal scintigraphy prior to surgery (Table 2).

Three patients had bilateral obstruction. Among the 6 operated lacrimal systems of these patients, 5 lacrimal systems with postsac obstruction had successful outcomes (83.3%); 1 lacrimal system with presac obstruction had an unsuccessful outcome (16.7%). All of the patients with unsuccessful outcomes declined further investigations and surgery.

## DISCUSSION

In the literature, there is inconsistency in terminology for describing patients with epiphora whose ducts are patent to nasolacrimal irrigation. Demorest and Milder^25^ first introduced the term ‘functional block’ in 1955 and described a case that was clinically patent to nasolacrimal irrigation and had an abnormal dacryocystogram (moderately distended lacrimal sac and retention of dye). In 1974, Duke-Elder and Macfaul^26^ described cases with patent systems as lacrimal insufficiency and included pathologies from punctal eversion to lacrimal sac tumours. In 1975 Hurwitz et al.^27^ described patients with ‘functional blocks’ as those having “epiphora and normal dacryocystograms”. In our study, patients were enrolled as FNLDO if they had epiphora with patent lacrimal systems and an abnormal lacrimal scintingraphy. Patients with pump failure, ocular surface disease or any other cause of hypersecretion and FNLDO due to systemic disease, medication or trauma were excluded.

Patients with functional obstruction of the lacrimal drainage system are difficult to diagnose. It appears that no standardized approach exists in the assessment of such cases. The results of Conway’s^21^ survey among the members (138 responses from 300 members) of the American Society of Ophthalmic Plastic and Reconstructive Surgery indicate that dacryologists favor different methods of assessment for this group of patients. In our literature review we had a similar impression; there appears to be different preferences in the clinical and radiologic assessment of this group of patients.^1,2,3,4,5,6,19,20,21,28,29,30^ The most preferred clinical tests according to Conway’s survey are nasolacrimal irrigation, primary and secondary dye test (Jones 1 and 2 tests) and fluorescein dye disappearance test (FDDT). Imaging with dacryocystography or dacryoscintigraphy may provide further information for diagnosis and management.^2,21^ In our study, nasolacrimal irrigation was performed as the first step of assessment in the tearing patient. In patients with freely patent lacrimal systems on nasolacrimal irrigation, our choice of radiological assessment was lacrimal scintigraphy. This procedure is easy to perform, non-invasive and also shows the location of the stenosis. In 1972, Rossomondo et al.^31^ introduced lacrimal scintigraphy, a radionuclide method of imaging the lacrimal drainage system that avoids intubation and allows a more physiological assessment of tear flow dynamics. In 1974, Chaudhuri et al.^32^ assessed the diagnostic accuracy of the lacrimal scintigraphy and dacryocystogram. They concluded that there was good correlation, although the scintigraphy was marginally superior. This statement was based on the dacryocystogram being unlikely to detect an abnormality in patients with FNLDO. However, in 1975, Hurwitz et al.^27^ reported that unless scintigraphy is used with quantitative analysis, it is of limited value and acts merely as a complementary test to dacryocystography. Wearne et al.^2^ stated that the main level of blockage in the lacrimal system was easier to detect objectively with lacrimal scintigraphy. The differentiation of abnormal lacrimal scintigraphies into presac, presac, or intraductal delays may provide help in clinical management and in predicting surgical success.^2,4^ In our study, we used lacrimal scintigraphy for diagnosis and location of the obstruction. The information we gained helped us determine whether or not to perform bicanalicular silicone intubation with ext-DCR.

There are several treatment options for FNLDO. Silicone intubation has been proven to be successful in adult partial nasolacrimal duct blockage; Angrist and Dortzbach^12^ achieved good results with this procedure in 74% of their patients with a partial obstruction. Moscato et al.^13^ reported the long-term success rates of silicone intubation as 96% at 2 years and 85% at 3 years, and approximately 50% of their patients had relief from epiphora between 5 and 6 years after silicone intubation. In a survey of the ASOPRS, 41% of respondents would treat a FNLDO with a DCR, while only 22% would use silicone intubation.^21^

Balloon dacryoplasty has patency rates of 25-50% at two-year follow-up.^14,15,16,17^ With the addition of silicone intubation and antegrade catheterization, Perry et al.^18^ documented patency rates of 73% with a shorter follow-up time of six months.

Ext-DCR is established as a highly successful procedure for complete stenosis of the nasolacrimal duct.22 However, its role in the surgical management of FNLDO is less predictable. Demorest and Milder^25^ suggested in 1955 that a radiographically demonstrated functional block in a patient with chronic epiphora is an indication for DCR and reported that patients respond just as dramatically as those with an anatomic blockage. Sahlin and Rose^24^ reported a retrospective study of patients with symptomatic epiphora and patent tear ducts, with at least two-year follow-up after DCR. In 50% of patients, they reported a marked improvement or cure of symptoms. O’Donnell and Shah19 demonstrated a 94% success rate for ext-DCR with silicone intubation in 51 FNLDO cases and their average follow-up duration was 9.6 weeks (range, 5-101 weeks). Peter and Pearson^3^ reported 63% success after an average of 11 months of follow-up for 46 ext-DCRs with silicone intubation in patients with epiphora and clinically patent lacrimal systems. Similarly, Delaney and Khooshabeh^4^ performed ext-DCR with silicone tube intubation in freely patent systems and reported their success rate as 84% (91% for postsac, 67% for presac delays) 3-4 months postoperatively. However, this rate declined to 70% (80% for postsac, 47% for presac delays) at 3 years. In postsac obstructions, we achieved a similar long-term success rate of 88.2% at 64.42 weeks average. However, unlike Delaney and Khooshabeh, we did not perform silicone intubation in these cases.

In our study we grouped our cases as presac and postsac obstructions according to lacrimal scintigraphy findings. We performed ext-DCR with bicanalicular silicone intubation in presac, and ext-DCR alone in postsac delay cases. Successful outcome was achieved in 76.9% (presac 55.5%, postsac 88.2%) of our cases. The aim of our surgery in presac obstructions was to overcome the inflammatory process in the canalicular system, while in postsac obstructions it was to decrease the distance from the puncta to the nasal mucosa. The inflammation in the proximal system is probably an ongoing process, leading to higher surgical failure.^4,17^

Our 76.9% overall success rate of ext-DCR for the treatment of FNLDO is markedly lower than our approximately 98% successful outcome of ext-DCR for primary acquired nasolacrimal duct obstruction. This relatively lower successful outcome in functional obstructions is probably because of the ongoing idiopathic inflammatory process responsible for the obstruction or other factors contributory to epiphora.^4,17,24^

There were some limitations in this study, such as the small number of patients. This prevented us from obtaining more reliable results. Another drawback of this study was the lack of postoperative evaluation with imaging techniques. We did our postoperative assessment on a subjective basis through patient complaints. These weaknesses should be considered in future studies.

In conclusion, our 76.9% success rate, especially 88.2% for the postsac delays, confirms that our approach is efficient for the management of patients with epiphora and clinically patent lacrimal drainage systems. For the postsac delays, ext-DCR without silicone intubation may be the choice of operation. Our average follow-up duration of 72.85 weeks is worth mentioning because it is longer compared to previous studies. Lacrimal scintigraphy was helpful in the preoperative assessment of our patients to determine the surgical approach-whether to place bicanalicular silicone tube or not-and to predict the surgical outcome, which was more satisfactory in postsac obstructions.

## Figures and Tables

**Table 1:**
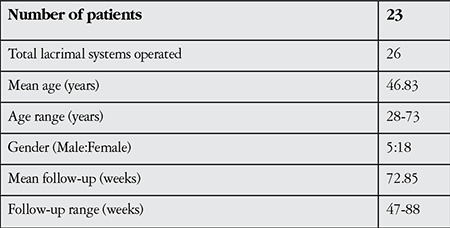
Baseline characteristics of the patients who underwent external dacryocystorhinostomy for functional nasolacrimal drainage obstruction

**Table 2:**
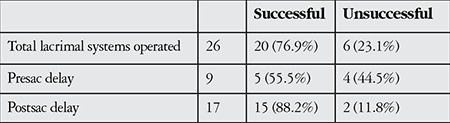
Surgical outcomes of external dacryocystorhinostomy in functional nasolacrimal drainage obstruction cases with regard to the location of the obstruction
